# Retraction: MicroRNA-367 promotes progression of hepatocellular carcinoma through PTEN/PI3K/AKT signaling pathway

**DOI:** 10.1042/BSR-20182466_RET

**Published:** 2020-03-13

**Keywords:** Hepatocellular carcinoma, MiR-367, PTEN, PI3K/AKT pathway

*Bioscience Reports* (2019); https://doi.org/10.1042/BSR20182466

The authors are retracting their accepted manuscript “MicroRNA-367 promotes progression of hepatocellular carcinoma through PTEN/PI3K/AKT signaling pathway” at the request of the corresponding author and Hunan Provincial Tumor Hospital, in conjunction with the policies of their Institutional Review Board (IRB), as their study had not been granted at Hunan Provincial Tumor Hospital. They had also noticed an error in terms in the institutional review board within their Declarations.

All the named authors of the original accepted manuscript have agreed to this retraction, and apologise for any inconvenience caused to the readers and to the Editor of *Bioscience Reports*.

